# Piloting a smartphone-based application for tracking and supply chain management of medicines in Africa

**DOI:** 10.1371/journal.pone.0217976

**Published:** 2019-07-17

**Authors:** Henry Omoregie Egharevba, Omolola Fatokun, Mercy Aboh, Olobayo Olufunso Kunle, Solomon Nwaka, Karniyus Shingu Gamaniel

**Affiliations:** 1 National Institute for Pharmaceutical Research and Development (NIPRD), Abuja, Federal Capital Territory (FCT), Nigeria (interdepartmental initiative); 2 Africa Network for Drugs and Diagnostics Innovation (ANDI), UNOPS, Addis-Ababa, Ethiopia; Public Library of Science, UNITED KINGDOM

## Abstract

A confounding factor for healthcare programmes in African countries is the inability of essential health tools to reach targeted locations and populations, due to poor Logistics Management Information System (LMIS). In a bid to contribute towards addressing these challenges, a pilot study was undertaken to evaluate the tracking ability, reliability and applicability of EASE App, a novel Smart Phone based Application. The App is designed to provide real-time tracking and tracing of commodities as well as curation of data in a cloud based database with restricted access which can be linked with other databases. In this study, NIPRIMAL was labelled with QR codes, and tracked within the Federal Capital Territory, Abuja, Nigeria, using the smartphone based EASE App. Data collected showed that the “EASE App” tracking had accuracy of 100% for date and time of scan, operators’ codes and product identity; and 92.83±1.69% and 99.83±0.27% accuracy for GPS mapping label for the city and country, respectively. The GPS mapping label for specific streets, roads or districts, gave an accuracy of about 64.28±3.14%. The technology was able to provide real-time data on user unique identity, user location as well as date/time of use, and the feedback report indicated that it was readily deployable and easy to use. The results demonstrate that the “EASE App” is a promising technology that can support supply chain and related data management challenges in resource poor settings. The potential benefit of the EASE App in strengthening LMIS and distribution chain system in Africa as well as future optimization of the App are discussed.

## Introduction

A viable distribution system relies on adequate and timely information on inventory, demography of targeted population, as well as availability of good storage and handling facilities at all strategic points of the supply chain. The system should be supported by a robust inventory monitoring and replenishment scheme to ensure uninterrupted supply and distribution. Hence, a comprehensive and effective Logistics Management Information System (LMIS), which ensures timely collection and reporting of information to various levels in the system to allow for effective decision making, is required [1–5].

This is not the case in most African countries where a large proportion of health programmes fail because of their inability to efficiently deliver health commodities to targeted populations. As a result, primary and secondary problems arise at the user end (such as hospitals or field locations), due to avoidable stock outs, over or under stocking, delays in distribution and delivery of health products. This in turn result in expiration during storage, wastage, wrong location deployment, counterfeiting, wrong use, pilferage, diversion, lack of accountability as well as lack of access to real-time data for informed decision making [1, 2].

A major challenge in establishing and implementing a viable supply/distribution system in Africa is the dearth of appropriate technology, necessary infrastructure and human resource capacity. Most African countries lack the technology and infrastructure base such as real-time movement tracing and tracking technology; appropriate digital data computing, processing and storage platforms/systems; and well-equipped warehouses or medical stores. This is coupled with the long distances to be covered in reaching communities that may not be readily accessible due to poor transportation network, among other challenges. These challenges put pressure on the limited human and financial resources, and create problems of delay in products delivery, poor inventory monitoring, opacity of operation and poor supply/distribution management. A system that guarantees whole-chain product visibility and easy access to real-time data in a readily analysable format will be a veritable tool for achieving an efficient distribution chain.

The manual non-digital or analogue based LMIS, such as the weekly dashboards [6], used by some African countries fail because of their cumbersomeness. More recently, digitalized LMIS tools such as electronic warehousing software, M-Supply [7], transport management system (TMS) [8], and other cloud based digital platforms like the District Health Information Software 2 (DHIS 2) [9], have been deployed in health care programs across Africa to monitor outcomes. The slow processing time and poor integration of data/information and analysis under these platforms result in ineffective LMIS. In addition, the data generated is usually lost or prone to abuse by unscrupulous persons due to unsecured access and storage. All these result in a weak distribution system and eventual programme failure in these countries.

Technological advancement in mobile cloud-computing systems and integrated wireless communication networks (3G/4G/5G), which has led to the big data stream mobile computing (BDSMC) infrastructure/system, has opened up new frontiers of potential application in solving the myriad of operational problems in healthcare delivery systems [10, 11]. These technologies have been optimized for health information exchange, personalized healthcare services, clinical diagnoses and electronic health records (EHR), through various electronic software and health-kits like Apple Health App, S-Health App, LG Health and DHIS 2 [9, 10]. Cloud-computing eliminates big data storage problems in mobile devices which usually lead to slow processing or response and high energy consumption. It has also eliminated slow and insecure access to data as well as reduced the capital /operational expenditure in data storage and access associated with non-cloud based systems. Though some researchers have proposed the integration of the cloud-computing and mobile network system into the transportation and logistics for enhanced lifestyle and city efficiency [8, 10–12], very little attention has been paid to its use in strengthening the supply/distribution chain management. A technology that could conveniently integrate the real-time cloud computing system, the wireless internet service and mobile devices in tracking/tracing health products from the manufacturer through the distribution chain to the end-user, will no doubt transform distribution systems. Such a technology should be simple, easy-to-use, able to track movement and use of health commodities even within the last-mile, importable and affordable through application of BDSMC infrastructure/system. The technology should also provide secondary or auxiliary benefits for monitoring drug misuse and counterfeiting.

As a result of a World Health Organization’s World Health Assembly supported demonstration project [13], and as response to the needs of healthcare delivery in Africa, the African Network for Drugs and Diagnostics Innovation (ANDI) collaborating with Ease-Medtrend Biotech and other partners initiated the implementation of an integrated smartphone-based App for health products tracking [14]. The technology was originally developed to boost the diagnosis of neglected diseases at point of care, and uses an Integrated Mobile Diagnostics Readout (IMDR), to obtain state-of-the-art results for multiple diseases in less time and at lower cost. The App, which is also known as the EASE App, was later optimized for health commodities tracking and tracing linked with cloud based data management.

The EASE App relies on available BDSMC platforms to enable wireless real-time energy-efficient data acquisition, transport and processing, allowing for variety (data heterogeneity), volume (continuously increasing amount of data to be processed or processed), velocity (fast rates of data generation), value (huge value but hidden in massive datasets at very low density), and volatility (real-time transportation and processing of acquired data stream) [11]. Key features of the technology include generic smartphone dependent QR (Quick Response) code reading; real-time GPS (Global Positioning System) location tracking; automated upload response alerts; unlimited data entries; high quality database; and multilevel data access control. The technology is able to capture, retrieve and upload encoded information, which is digitally processed at a speed of about 2 seconds per capture and stored in a remote central server (cloud). This information can be viewed and shared using appropriate digital platforms, or further linked to other databases in an integrated manner. This technology is particularly suited for Africa where most countries have problems with provision of adequate infrastructure for huge data processing, transmission and storage or archiving. It will also reduce the challenges of lack of reliable health statistics in some African countries, especially in sub-Sahara Africa.

In this study, we demonstrate the utility of the EASE Smartphone Application in supporting the medicines distribution chain, data management and prompt LMIS decisions in Africa. We also identify features of the App that require optimization for improved performance and better data analysis.

## Materials and method

### Materials

NIPRIMAL product and labels were provided by the National Institute for Pharmaceutical Research and Development (NIPRD), Android based smartphones were provided by study volunteers, the EASE App (downloaded and installed from web-link) as well as serially coded QR codes were provided by Ease-Medtrend Biotech (EMB) Shanghai China. Other materials, which include Laptop computers, printer and internet modem, were procured locally in Abuja.

### Method

#### Pre-project activities

Recruitment of Participants: Twenty-five volunteers were initially recruited from the NIPRD community including Staff, Pharmacists interns, and students on industrial work experience scheme. The intention was to select twenty volunteers from the pool of twenty-five to be issued the twenty predetermined operators’ unique codes or login details. The number of operators’ unique codes was set at 20 to give a ratio of 10 products to one operator. Initial selection of volunteers was based on the possession of Android phone, EASE App compatibility with phones and spread of residence location within the FCT (for geographic spread). Compatibility was determined by ability to successfully download, install and subsequently login with the App. The targeted twenty volunteers were enlisted in the project from those that passed this stage. The volunteers were then trained for two days, at the end of which, they were able to install the EASE App on their Android phones, login with username and password, perform a scan of QR code and ensure successful upload of tracked data. Feedback forms were designed for the participants to rate field operation experiences on login, scanning, upload internet strength and number of trials before capture and upload, as very easy (VE), easy (E), good (G), fair (F), difficult (D), Varies (V), poor (P), couldn’t upload (CU), couldn’t scan (CS), and not easy (NE), in order to determine the ease of deployment of the Ease App.

Attachment of QR codes to product labels: The two hundred (200) QR codes received from EMB, were carefully detached and attached to product labels with the aid of a translucent liquid gum. Attachment of QR code took about an hour with two persons working together. Majority of the QR codes were scanned minimum of 3 times, with some scanned 6–8 times for demonstration/confirmation purposes.

#### Field activities

Product tracking: The QR codes on the labels were scanned on arrival at NIPRD store using specified operator login details, and subsequently issued to study participants to take to their residents and other locations that they frequently visited outside NIPRD. The operators scanned the QR codes at least three different times and/or in three locations using specifically assigned operator login information. The products were returned to NIPRD store after twenty-four hours and successful scanning. No personal identifying information was collected from the volunteers directly or through the EASE App. The only identifying information noted for the purposes of analysis was operator’s code (unique login details) assigned to the volunteers in the course of the study. The data collection process and subsequent use had no impact on the delivery of drugs to repositories or patients, and complied with the terms and conditions of use of the EASE App as provided by its developers, EASE Medtrend Biotech Limited. The exercise lasted eight days and the following primary outcomes were determined as percent accuracy within ≤1km radius, which adequately covers GPS coverage of the last mile of the distribution chain. The expected accuracy for all the tracking parameters was set at 95±1.43%.

- Accuracy of Location Specificity: (i) Number or % scan/QR codes indicating Nigeria (ii) Number or % scan/QR codes indicating Abuja, (iii) Number of scans/QR codes indicating the specific Street or District within Abuja.
- GPS accuracy: This refers to the number of scans indicating accurate GPS location as indicated by physical positioning during scanning within a radius of ≤1km.
- Time/Date: This refers to the number of scans showing accurate date and time of scanning.
- Operator details: This refers to number of scans showing the correct operator’s code.
- Other analyses and discussion focused on area distribution of product tracked outside NIPRD, strength of internet connectivity at location of scan, ease of login, scanning and upload, and number of trials before success, based on participants’ feedback.

The metric used was “percentage accuracy” (0–100%). Generally, the definition ascribed to “success” throughout the manuscript was based on expected positive outcome of the data to be captured, which include accuracy of location specifying name of area (country, region, district and street), time of upload, products information as contained in the QR code, and operator’s identity. With reference to location, success meant the data captured in the database corresponded with the exact physical location up to district name and street.

#### Statistical analysis

Statistical analysis was done using statistical tool SPSS 19 and Microsoft Excel tools on Excel spread sheet.

## Results and discussion

The statistical analyses of the tracking study in percentages are depicted in Table 1. The table shows mean (average) scan per QR code, the App’s tracking and tracing response for dates and time of scan, and location label for Country, State and districts/area/streets or road at the time of scan.

**Table 1:** Results of tracking of Niprimal using the EASE App.

S/N	Item / Parameters	Value
1	Mean Scans/QR code	4.47±1.37^a^
2	% Accuracy of scans showing Nigeria	99.83±0.27^b^
3	% Accuracy of scans showing Abuja	92.83±1.69^b^
4	% Accuracy of scans showing Specific Streets/roads /district	64.28±3.14^b^
5	% Accuracy for Date / Time of scan	100.00±0.00^b^
6	% Accuracy for operator’s Identity (unique code)	100.00±0.00^b^

^a^ standard deviation

^b^ margin of error at 95% confidence limit

Sample size used in computation includes the total number of uploaded data which was 893

### Location specificity

**Fig 1:**
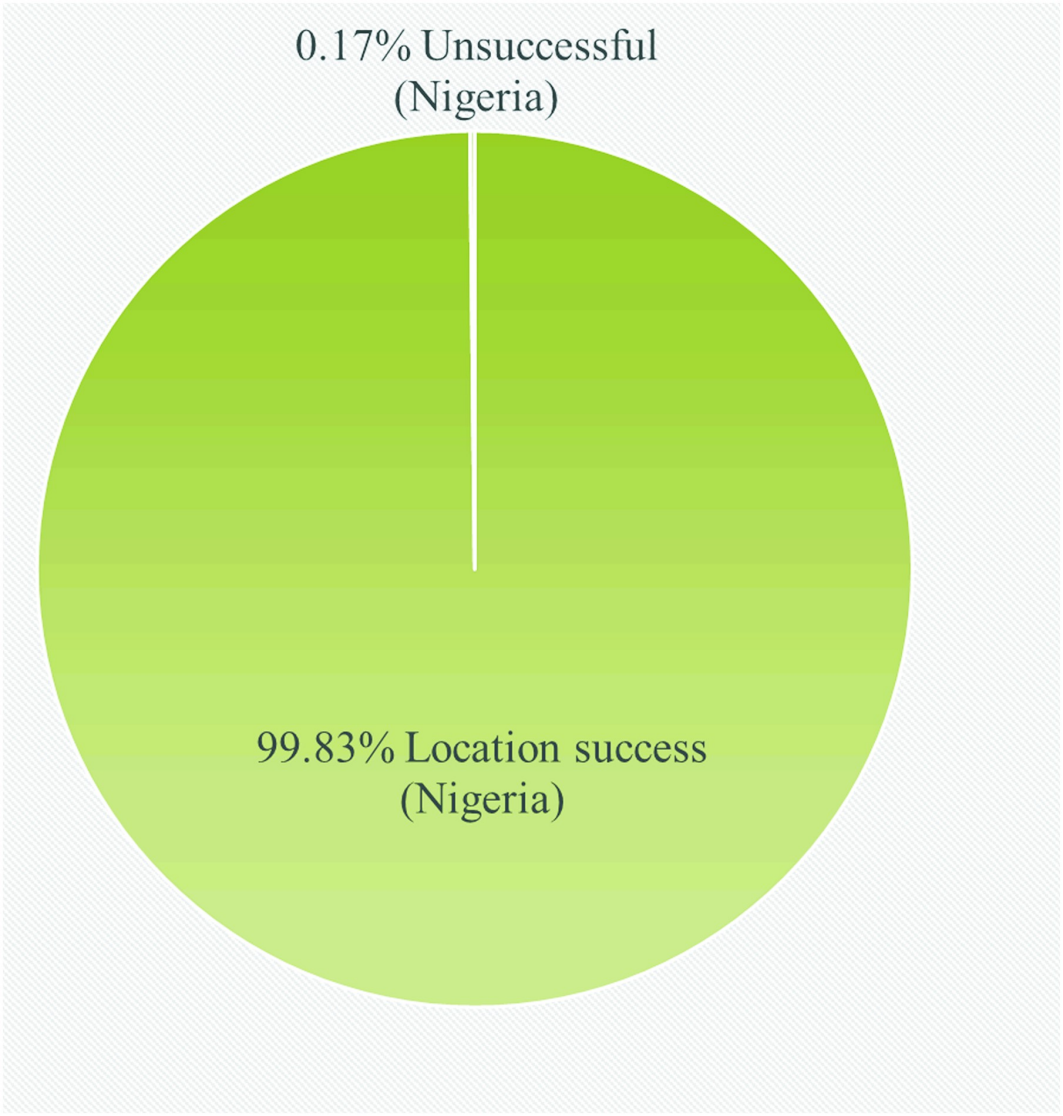
Chart showing the proportion (as %) of scans that correctly identified Nigeria as location and those that did not.

**Fig 2:**
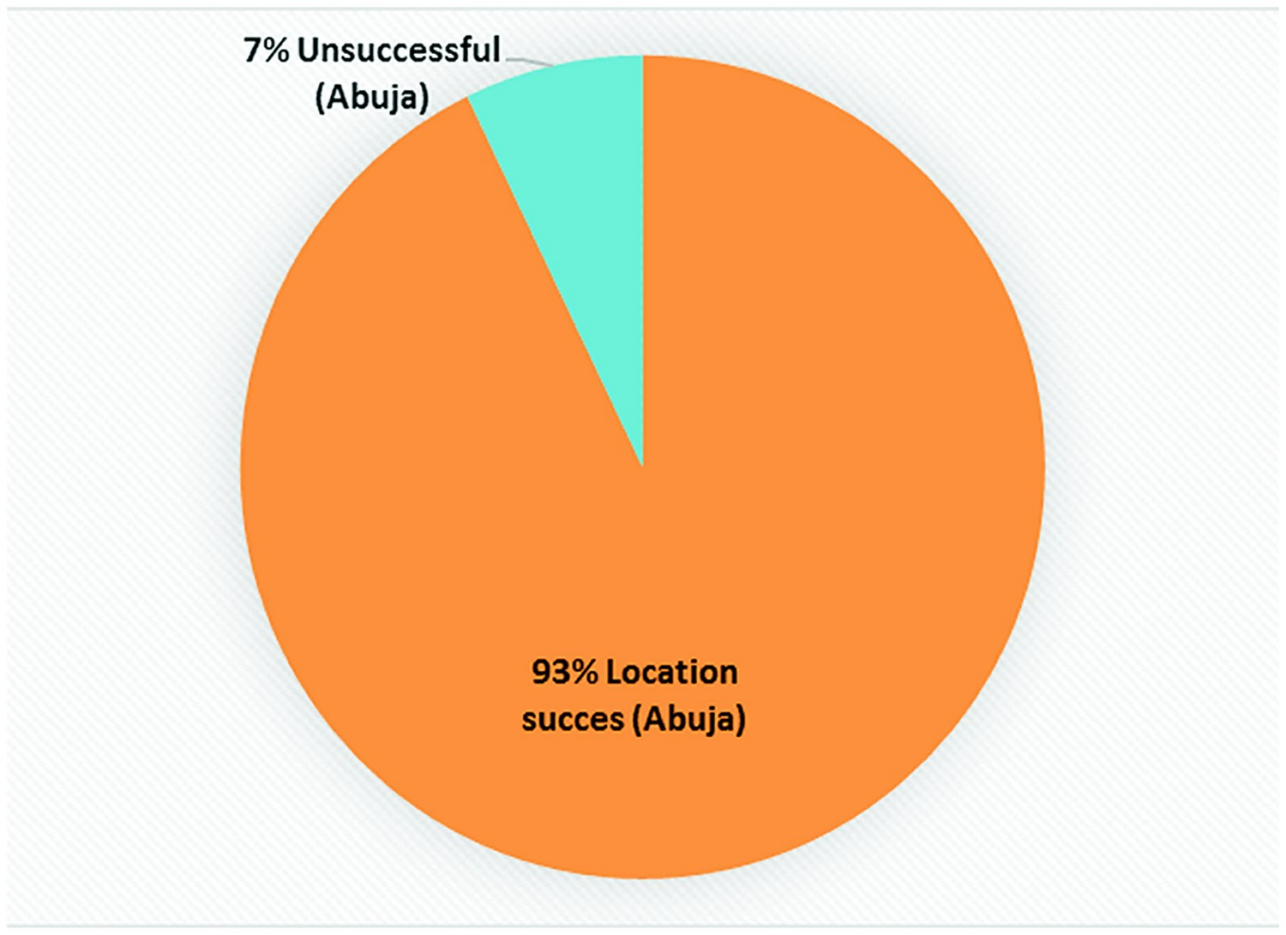
Chart showing the proportion (as %) of scans that correctly identified Abuja as location and those that did not.

The accuracy of scans reflecting Nigeria in the GPS mapping label was 99.83±0.27% (Fig 1), while 0.17% incorrectly specified India and China as the country location. This represents a minor country GPS label failure. Only 7.17±1.69% of the scans for State (Province) failed to correctly identify Abuja/FCT as the study location (Fig 2). Some of the scans with incorrect location label displayed GPS locations like Lagos and Port Harcourt, which are States/locations in Nigeria that are over 500 km from the study site. The results indicate excellent country and state/city specificity. City specificity was significantly lower (p<0.05) than country specificity (Fig 2) probably due to poor internet connectivity and or usual GPS errors due to attenuation in satellite signals or GPS constellation protocol [15, 16].

**Fig 3:**
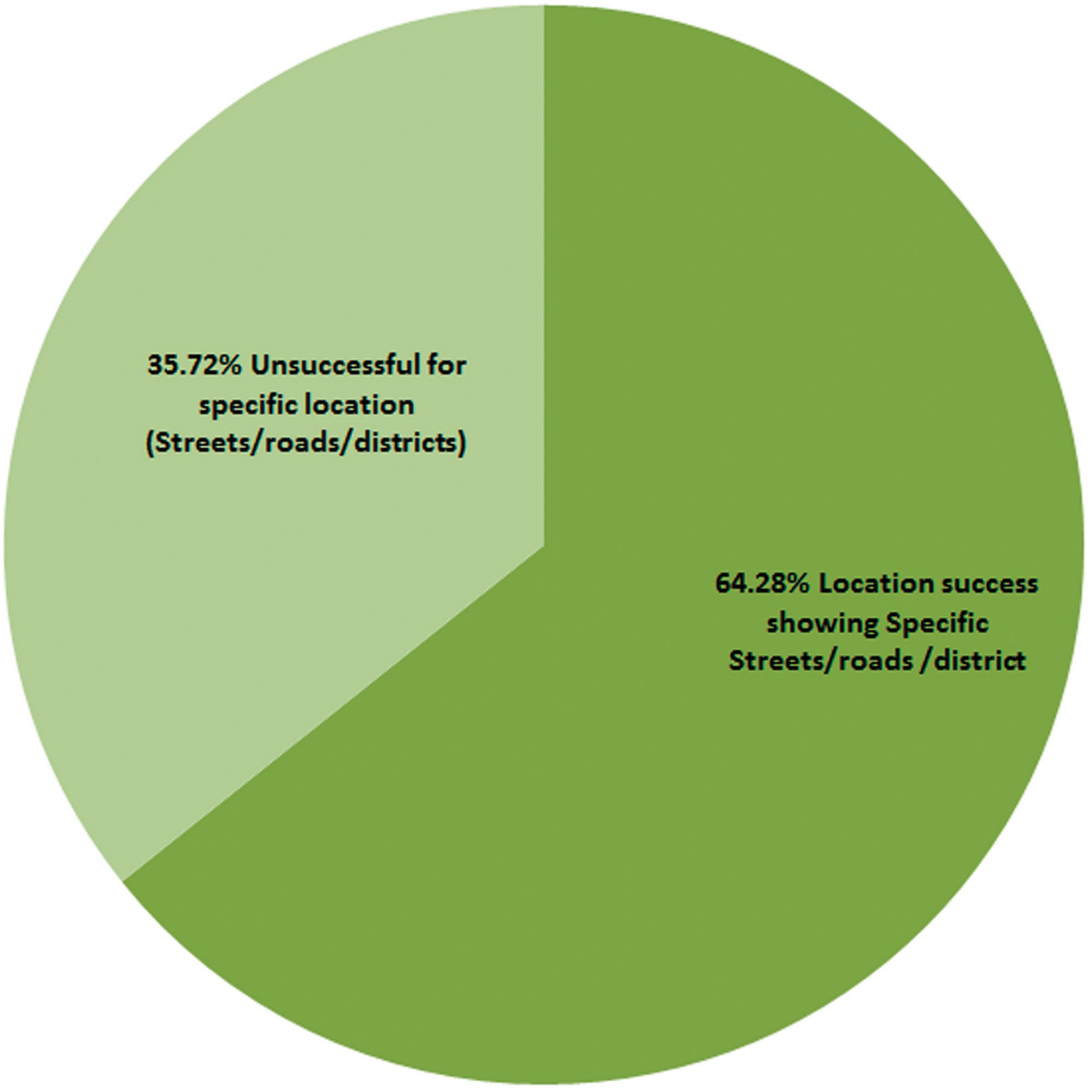
Chart showing the percentage success and failure of scanned QR codes showing specific location (streets/roads/districts).

Fig 3 shows GPS location specificity based on exact streets, roads and area in Abuja. The GPS label for streets/districts displayed by scans was 64.28±3.14% accurate, which was low compared to the results obtained for country and Abuja city, and lower than expected accuracy. Approximately 35.72±3.14% of GPS tracked locations were either not identifiable within a 1 km radius in Abuja or displayed “unknown road Abuja”. GPS location labels showing locations outside Abuja were also included in this category.

**Fig 4:**
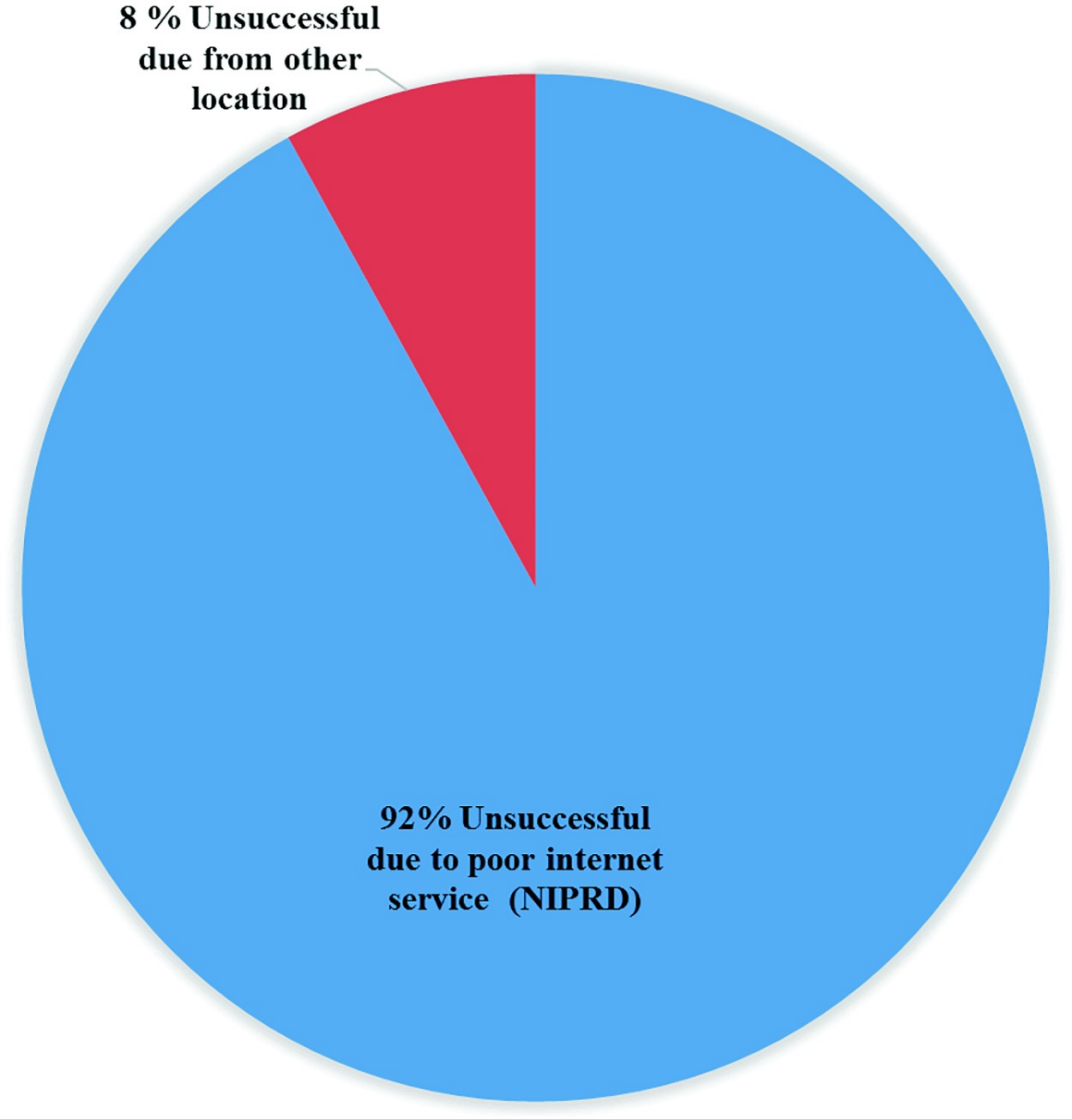
Chart showing the percentage location failure due from NIPRD and other areas outside NIPRD (combined).

Of the 319 (35.72±3.14%) scans that failed to show specific street/road/district, the ones carried out at NIPRD store, which was the start and end points in the distribution chain for this study, were responsible for 292 (91.54±3.05%) as depicted in Fig 4. NIPRD is located in Idu, a remote satellite area of Abuja, consisting largely of rural settlements. As a result, the strength of the internet signal and GSM connectivity is generally poor and unstable in this suburb compared to the better developed areas of the metropolis like Asokoro, Maitama, Wuse and the Central Business District. The other 27 (8.46±3.05%) failed scans came from Efab Estate—Life Camp 14 (4.39 ±1.34%), Bwari 7 (2.19 ±0.96%), Jabi 2 (0.63±0.52%), Garki 2 (0.63±0.52%) and Orozo 2 (0.63±0.52%) which are locations outside of the Abuja City centre. The failures at NIPRD and Efab Estate were generally attributed to poor internet connectivity/strength reception at the location and time of scan. Since signal reception is usually significantly weaker indoors in areas with poor internet connectivity, carrying out the scans indoor, as was the case in this study could be a reason for the low street/road/district specificity observed. The feedback from the operators (through feedback forms) showed that some of them experienced some level of difficulty in scanning and uploading the QR codes due to poor internet connectivity/reception in their locality. Since the App is most likely to be used indoors, it is recommended that further development efforts be focused on improving its sensitivity to internet signals to improve connectivity, or an Android phone model with systems that enhance positional accuracy such as Assisted GPS (AGPS), which may require a general packet radio service (GPRS), better internet sensitive and connectivity be used [17, 18].

The location failures could also be because the EASE App, which does not display GPS coordinates, relies on pre-captured satellite-dependent Google maps for the identification of locations. In addition to this, problems with the device/App and constellation system/protocol could also create GPS errors (ephemeris error, troposphere and ionosphere delay errors, receivers’ dependent errors, point selection and multipath errors, etc.) [15, 16, 18]. An improvement on the App to capture coordinates along with Google map label might resolve this challenge. Capturing of GPS coordinates will provide the tool for re-verifying suspected failed locations labels from Google maps since mislabelling is a known phenomenon on Google maps. For instance, where the labels showed Lagos or Port Harcourt, GPS coordinates could have been used to verify these location labels by means of a Google search.

### GPS accuracy

There was substantial failure (approx. 36%) in identifying the specific street/road or district/area within the Abuja study area. However, analysis of identified streets/road/districts with physical location or places scanning were performed gave an accuracy of 100%. As discussed earlier, failure would have been greatly reduced or even eliminated through coordinates search if the App provided for GPS coordinates capture along with the GPS labels. No association could be established between factors such as model of smartphone or the picture resolution quality of the phone camera used and GPS accuracy.

**Fig 5:**
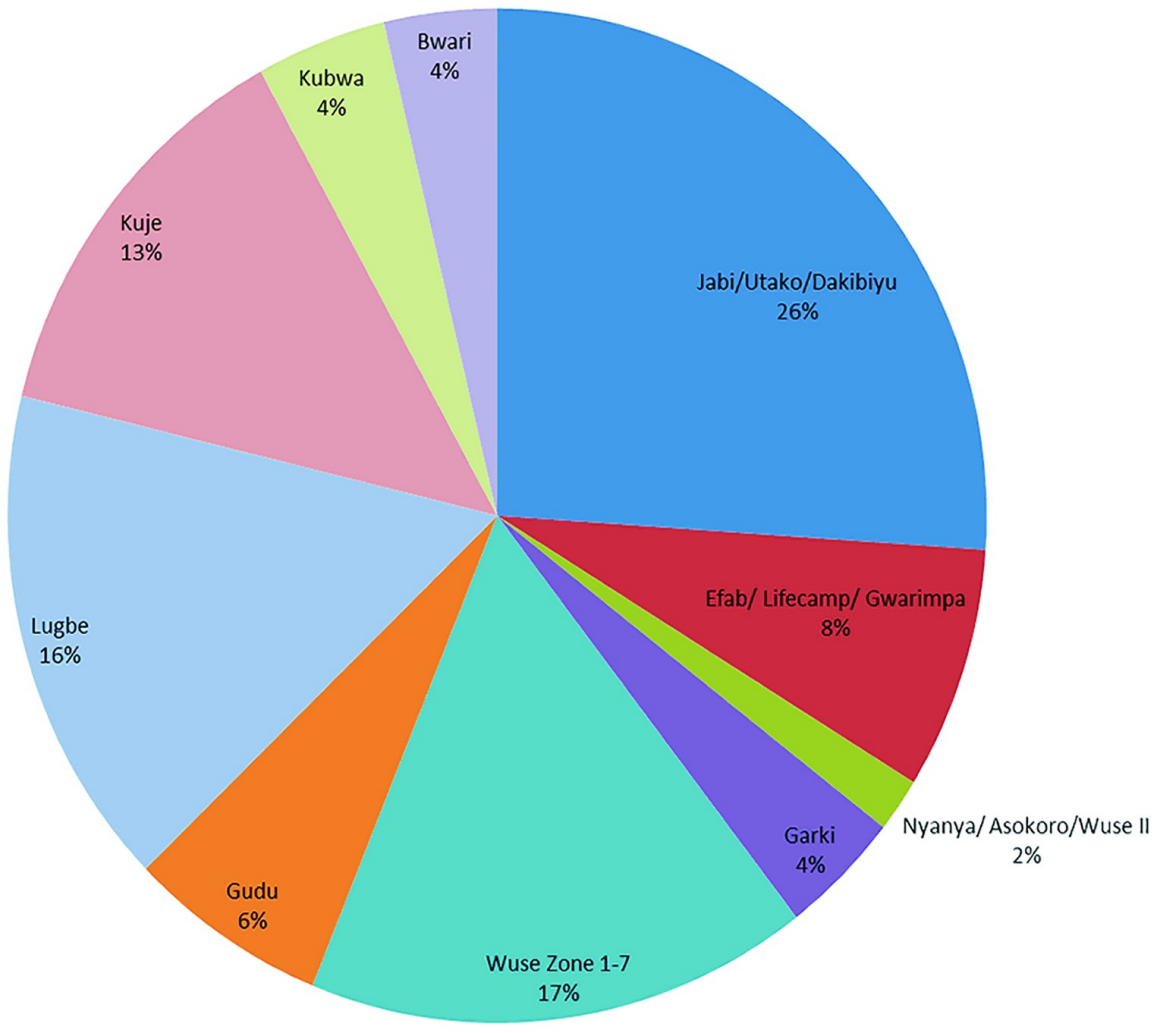
Tracking traceable to known areas (districts) in Abuja and environ excluding Idu/Karmo area (location of NIPRD).

Fig 5 shows the spatio-temporal distribution of the tracked locations of the medicines in the course of the study. If this is extrapolated to mean the quantity of products, the chart can be used to deduce product location. This would make the EASE App a useful tool in planning general logistics and monitoring of medicines distribution.

### Accuracy of date and time of scan and operator’s identity

The date and time of capture as shown in the data retrieved from the cloud completely corresponded with that obtained at the time of physical capture during the field work, with an accuracy of 100% (Table 1). The uploader data also correctly captured the specific operator’s unique code. This correspondence between these sets of data can provide an indication of operator (field worker) activity or operational efficiency. It could also be used by analysts or monitors to determine the rate of products’ use and the actual time of use, for materials reconciliation or reports validation.

### Ease of deployment

The descriptive statistic (Table 2) of the feedback reports obtained from the participants showed that none of them reported difficulty or inability to login; 61.5±18.3% and 38.5±18.7% reported very easy and easy, respectively. For scanning, 53.8±19.2% and 34.6±18.7% reported easy and very easy, respectively. But 7.7% and 3.8% reported not easy and could not scan, respectively. 43.6±19.1% and 30.8±17.75% reported easy and very easy respectively for ability to upload after scanning, while 20.5% and 5.1% reported difficult to upload and could not upload, respectively. This gave an easy of upload verdict of 74.4%. On number of trials before success, only 61.5±18.3% was able to achieve complete overall success on first attempt, 17.3% on second trial attempt and 11.5% on the third trial. About 3.8% reported failure. The failure reported was simply an inability to complete the entire process from login to upload. Since there was no report of inability to login, it was assumed that the failure may have occurred at the scanning and upload stage, which requires internet connectivity. 61.5±18.3% reported good network connectivity, 30.8±17.8% reported fair connectivity while 7.7% reported poor connectivity. This gave 92.3% for fair to good internet connectivity. However, 100% of those that reported poor internet connectivity also reported difficulty and failure in upload. Those that reported fair internet network either reported multiple trials or difficulty in scanning and upload. The feedback report indicate that internet connectivity is a primary requirement for the Ease App as it currently is and an improvement would be necessary for internet connectivity deficient areas.

**Table 2:** Field feedback report.

Scoring Criteria	% Feedback Report				
	Login	Scanning	Upload	Number of Trials	Internet connectivity
**VE**	61.5± 18.3	34.6± 18.7	30.8± 17.8	-	-
**E**	38.5± 18.7	53.8± 19.2	43.6± 19.1	-	-
**G**	-	-	-	-	61.5± 18.3
**F**		-	-	-	30.8± 17.8
**D**	-	-	20.5± 15.5	-	-
**V**	-	-	-	-	-
**P**	-	-	-	-	7.7 ± 10.3
**CS**	-	3.8± 7.4		-	-
**CU**	-	-	5.1± 8.5	-	-
**NE**	-	7.7 ± 10.3	-	-	-
**1**	-		-	61.5± 18.3	-
**2**	-		-	17.3± 14.5	-
**3**	-		-	13.4± 13.1	-
**≥4**	-		-	3.8 ±7.4	-

Key: Very easy (VE), easy (E), good (G), fair (F), difficult (D), Varies (V), poor (P), couldn’t upload (CU), couldn’t scan (CS), and not easy (NE)

Twenty six feedback reports were received and used in the analysis.

From the feedback analysis and data available in the cloud, all the QR codes were scanned within the period and time specified in the study design. All the product labels were promptly scanned by the operators within two hours of receipt of the medicine. Scanning and data upload at locations with good internet service took less than 2 seconds. There were reports of delay or failure of scanning and upload at certain locations attributable to poor internet data connectivity. From the feedback analysis, the smartphone-base Ease App is easily deployable in areas with strong internet connectivity. The effect of poor location label can also be minimised by the availability of operator’s details and time of upload. Since operator’s login is unique to each operator, it could provide additional information for confirming location.

### Cloud data

Uploaded data was immediately available on the tracking list in the App and could be viewed by anyone with authorised access. This provides an efficient monitoring platform that could be used to track operators’ activities, promote accountability and ensure effective audit. The cloud data included the name of product, manufacturer, lot number, index or serial number and the tracking details. The tracking details included the date, time and location of use (where the QR code was scanned), and operator code. The data was also available in the form of excel spread-sheet when login directly through the website (http://data.wosoft.me) rather than through the App. However, the format did not allow for easy query and the sequential (or customised) organisation of data in terms of location, specific time or date, product’s name, lot number, operator’s identity, etc. An improvement of the App would be required to resolve this gap to make it more versatile.

This study has revealed a 100% tracking reliability for date and time of tracking as well as operator’s unique identity for the EASE App. The GPS mapping label accuracy of the App was 99.83% for country and 92.83±1.69% for city. The specific location tracking accuracy was a relatively lower 64.28±3.14%, most probably due to poor internet connectivity. This however may not be a major drawback since main distribution chain nodes (such as major stores or storage facility) would be equipped with good internet data service to facilitate ease of data upload. The effect of low specificity due to poor internet connectivity can further be minimised by the availability of operator’s details and time of upload which had 100% accuracy.

Based on our analysis, we summarise areas where the App would benefit from further optimization:

1. The GPS tracking tool in the App could in addition to utilising the Google map location labels, capture the GPS coordinates, which would be helpful in verification where the street or district name is either unknown, unavailable or in doubt.
2. Organisation of cloud data should be such that the index numbers of the QR codes for a particular manufacturer or lot are arranged sequentially for ease of access.
3. A tool could be provided on the cloud platform or website to allow for data to be queried based on location, date or time of upload; or Manufacturer/lot number, QR code index number/operator’s identity to allow for easy data analysis.
4. The EASE App could be supported with short message service (SMS) for areas with poor data service. Such SMS should be able to register or decode location of origin or nearest base-station.
5. Optimization can include the ability for local storage of captured data including GPS coordinates on the smartphone memory which can automatically upload to the cloud database when there is internet service.
6. The App should be optimized to automatically return to scan mode (within 3 seconds) after upload is completed, or a menu tool provided on the upload page that could take a user to the scan mode when clicked or prompted rather than the tracking list page.

The use of QR coding technology is not new and, most currently available QR code tracking technologies use non-portable stand-alone computers, scanners and localized databases for local storage and order placement or dispensing facility [19, 20]. Most of these technologies however, do not operate on a central server or with portable cell phone. Additional benefits of the new technology include: real-time data gathering, database enrichment and management; drug information service; health commodities quality monitoring; tracking of precise location of product; supports for improved Logistics; transparency and accountability of distribution chain workforce. This type of technology will support the attainment of the sustainable development goals [21, 22].

Finally, establishing a good distribution system or structure is only possible through real-time data access for forecasting, planning and standard logistic management information system (LMIS) which the EASE App provides. The study has demonstrated ease of operational deployment due to the App’s compatibility with low-cost Android smartphones, 3G/4G internet network services, ease of personnel training, and its interactive nature. The tracing and tracking capabilities of the App coupled with its efficient deployment could lead to reductions such as losses due to expiration, over/under stocking and poor accountability. The technology is expected to run on existing human and physical structures which would result in further reduction in the size of capital and operational expenditure of the existing supply/distribution chain management infrastructure. Our results show that the “EASE App”, is a promising technology that can be used to support supply chain and related data management in resource poor setting.

The study was however constricted by the fact that only a section of the supply chain was covered. The study was restricted to the last mile distribution and not the entire chain because, the last mile distribution is the most challenging in the chain, and if the technology works well in the last mile distribution, it is expected to work well throughout the chain. Subsequent studies will cover the entire chain and include a comparison to similar tracking devices to determine sensitivity, and degree of performance in simulated and real situations. Such study may deploy pre-calibrated smartphones updated with GPS coordinate and synchronised Google maps capabilities to eliminate errors due to non-uniformity across mobile devise.
